# The Non-Malarial Fevers of the Tropics

**Published:** 1901-02-02

**Authors:** 


### THE NON-MALARIAL FEVERS OF THE TROPICS.

As one result of the growing feeling of certainty in regard to the mosquito theory of malaria, and of the general recognition of the reliability of the indications given by the microscope in the diagnosis of the disease, it is probable that we shall before very long find a large increase in the number of separate and well-differentiated febrile maladies met with in the tropics. Wherever any one particular disease greatly predominates there is a natural tendency among those who practise in that district to attribute to the predominant ailment any malady which has even the most superficial resemblance to it, and this has been markedly the case in regard to malaria. Malaria often presents anomalous features, and in malarious countries it is frequently found that strange and obscure cases clear up under quinine. Hence the strong temptation to dub every fever malaria and every case of debility malarial cachexia; a temptation to which one can hardly doubt that many practitioners have given way. The result has been, on the one hand, much loss to many patients by time being wasted in unavailing efforts to cure them with quinine; and, on the other, much undeserved doubt as to the efficacy of quinine in consequence of this drug being used in cases to which it was quite inexplicable. There can be but little doubt that among the crowd of fevers which have been spoken of as abnormal cases of malaria, or explained as double infections overlapping one another, or united together as malarial remittents, a large number are not malarial at all. The efficacy of quinine has had the effect of demoralising the doctors in malarious districts, and this drug has seemed sometimes to have become the alpha and omega of all medical effort. Signs are not wanting, however, that the efforts of the large number of well-qualified men who are now engaged in the investigation of tropical fevers—and are now able, armed with the microscope, to separate malarial from non-malarial fevers—will before long throw considerable light upon this very large question. By aid of the power they now possess of putting on one side most cases of malaria, they will be able to devote themselves to the task of differentiating the various diseases which make up the remainder, and we can hardly doubt that before many years have passed the subject of tropical fevers, hitherto so dominated by the ever-present intrusion of malaria, and obscured by the routine treatment by quinine will have to be entirely rewritten.

